# Pectinase from Microorganisms and Its Industrial Applications

**DOI:** 10.1155/2022/1881305

**Published:** 2022-03-11

**Authors:** Setegn Haile, Abate Ayele

**Affiliations:** Department of Biotechnology, College of Biological and Chemical Engineering, Addis Ababa Science and Technology University, 16417 Addis Ababa, Ethiopia

## Abstract

The utilization of microbial pectinase in different industries has been increased in its world demand. The major sources of pectinase are microorganisms mainly bacteria, fungi and yeast. The utilization of low-cost agro-industrial wastes as substrates has been preferable in pectinase production. Pectinase production faced various parameters optimization constraints such as temperature, pH and production times which are the main factors in pectinase production. The pectinase enzyme is getting attention due to its several advantages; hence, it needs to be explored further to take its maximum advantage in different industries. This review discusses the pectin substance structure, substrate for pectinase production, factors influencing pectinase production, the industrial application of microbial pectinase and also discusses challenges and future opportunities of applying microbial pectinase in industry.

## 1. Introduction

Enzymes are substances present in the cells of living organisms in small amounts which are capable of speeding up chemical reactions without themselves being altered after the reaction. As compared to chemical catalysts, enzymes have many advantages such as high specificity, a high catalytic efficiency, and an adjustable activity, which greatly promote the use of enzymes in pharmaceutical, chemical, and food industries [1, 2]. Due to these desirable features, the demand for industrial enzymes has catapulted to new heights which call for constant research and development, to optimize their production and minimize resource costs [3]. The discovery of enzymes was started in the middle of the nineteenth century and they were first introduced in the industrial application using fungal enzymes. However, after twenty years Boidin and Effront introduced the bacterial enzymes in the industry [4].

Most of the industrial demand for enzymes is originated from microorganisms. Due to their high growing capability, short life span, and easiness of genetic manipulation, microorganisms are preferred in industry for enzyme production. Microbial enzymes are supplied, well-standardized, and marketed by a few competing companies. Among these industrially important enzymes, pectinases have a special significance due to their multiple uses in important sectors such as food, textile, beverages, pulp and paper, and biofuel industries [5]. The microbial pectinases account for twenty-five percent of the worldwide food and industrial enzyme scale and market increase from time to time [6].

The commercial applications of pectinases were observed in the 1930s [7, 8]. Pectic enzyme is another name of pectinases that belongs to the polysaccharides family [9]. They assist plants in their cell wall extension, tissues softening at the time of maturation and storage and also ecologically maintain the plant's waste materials decomposition and recycling [10]. Pectinase enzymes are extensively used in the food industry particularly in fruit juice extraction and clarification [11].

The aim of this review begins with an overview of pectinases, their structure, and pectic material classification. Factors affecting pectinase production, pectinase substrate, pectinase microorganisms, and pectinolytic substrate are also addressed, as well as numerous industrial applications of microbial pectinases and challenges and future opportunities for using microbial pectinase in industry.

## 2. Pectic Substance and Its Structure

**Figure 1:**
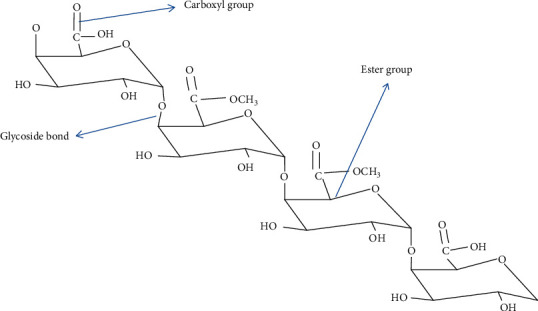
Structure of pectin and its functional groups [14].

The compound hydrolyzed by pectinase has generic names called pectic substances. Pectin is also defined as a polysaccharide that is rich in very important sugars, galacturonic acid and methanol as main components and has a high molecular weight heterogeneous and acidic structural polysaccharide which is one of the major ingredients of cereals, vegetables, fruits and fibers [12]. Pectin constituent of the middle lamella and primary cell wall in the plant cell wall and within the wall forms a matrix in which a network of cellulose and hemicelluloses. According to Hassan and Ali [13]; the pectic substances are complicated colloidal acid polysaccharides with a long galacturonic acid pillar chain and interconnected together by glycoside bonds in Figure 1. This is the basic unit of pectic substances which is also known as homogalacturonan.

Pectin is one of the most complex bio-macromolecules in nature and it can be composed of 17 different monosaccharides and at least seven different polysaccharides as reported by [11]. Nighojkar et al. [15] reported that other sugars, such as D-glucuronic acid, L-fucose, D-glucose, D-mannose, and D-xylose are found in the side chains of pectin substances. Based on the American chemical society pectic substances that are used as a substrate in the pectinase productions are classified as pectic acid, pectin acid, pectin and protopectin. The main important criteria to classify those pectic substances were based on the solubility of these pectic substances by water in Table 1 [16].

Pectin is considered one of the most complex substrates (heteropolysaccharides) found in the cell wall and middle lamella of fruits and vegetables [17, 18]. Pectins have been detected in fruits and vegetables such as apple, citrus and beet in Table 2.

### 2.1. Pectinase and Its Classification

Pectinases are defined as mixed enzymes that hydrolyze pectic substances, mostly present in microorganisms and higher plants [11]. Pectinase is the collection of enzymes that catalyze the pectin-containing substances produced by plants and microbes. The majority of commercial enzymes are obtained by employing fungal cultures [20]. Pectinase is involved in the metabolism of the cell wall as well as in the growth of the cell, senescence, ripening of fruits, pathogenesis and abscission process. Pectinases have been commercially prepared from the microorganisms especially from the fungi since the 1970s′, [21]. According to the enzyme commission and the international union of biochemistry, pectinase enzymes are classified under the hydrolase group [22].

#### 2.1.1. Protopectinase

As explained in the previous Table 1, the insoluble part of pectic substances is called protopectin and it needs other solvents to degrade pectin. Protopectinase is one classification of pectinase that degrade insoluble protopectin which is present in unripe fruits and gives rise to highly polymerized soluble pectin. The other name of this enzyme called pectinosinase [23]. Hassan and Ali [13] reported that protopectinase breaks down the insoluble protopectin into highly polymerized soluble pectin and its activity is can be analyzed by carbazole sulphuric acid method with the help of which the amount of pectin related material released from protopectin can be determined.

#### 2.1.2. Pectin Methylesterases

One of the main functional groups in the pectin structure is methoxyl ester as shown in Figure 1. Pectin methylesterases are responsible for the removal of this methoxyl group from pectic substances, and this removal process finally produces pectic acid and methanol. Pectin methylesterase is also known as pectin pectylhydrolase, pectinesterase, pectin demethoxylase, pectase, and pectolipase, is a carboxylic acid esterase and belongs to the hydrolase group of enzymes [24].

**Figure 2:**
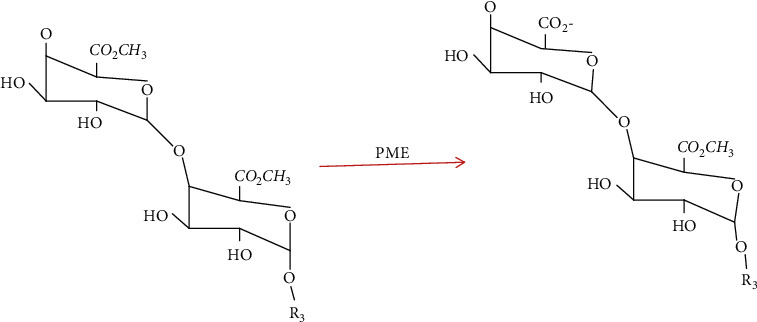
Action of Pectin methylesterase on polygalacturonic acid chain [13].

Pectin methylesterase which is extracted by fungi species act as a multichain system in the removal of a methyl group in randomly action and if pectin methylesterase produced by plants is acting either at a non-reducing end or next to a free carboxyl group and continue along the molecule by a single mechanism [25]. Pectin methylesterases de-esterification of methyl ester bond at *α* 1–4 *D* galacturonosylsubunit with the addition of water and produces Pectate polymer which is negatively charged and methanol in Figure 2 [26]. As Oumer [14] reported, the action of pectin methylesterase in pectic substances finally produced pectate.

#### 2.1.3. Polygalacturonase

**Figure 3:**
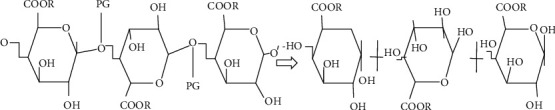
The action of polygalacturonases on polygalacturonic acid chain [13].

Polygalacturonase one of the classification pectinase and is also called depolymerase because it acts in the depolymerization process. Polygalacturonases are the pectinolytic enzymes that catalyze the hydrolytic cleavage of the polygalacturonic acid chain in the presence of water [26, 27]. Therefore, the function of this enzyme is splitting the alpha 1–4 glycoside bond between galacturonic monomers. Patidar et al. [28] investigated that polygalacturonases are categorized into two parts. Those are endo-polygalacturonase and exo-polygalacturonase. Endopolygalacturonase hydrolyses polygalacturonic acids and liberates oligogalacturonic acids. Exo-polygalacturonase hydrolyzes pectic acids and liberates mono-galacturonate. Polygalacturonases are the pectinolytic-depolymerase enzyme that hydrolyses polygalacturonic acid chain at the point of 1⟶4 *α* glycosidic linkage with the induction of water molecule in Figure 3. Polygalacturonasesare most widely studied in the family of pectinase enzymes that have functional, technical and biological involvement in the industries processing food and in the interaction between plants and fungus [21].

### 2.2. Substrate for Pectinases Production

Phutela et al. [29] reported that the natural substrates like malt sprout, wheat bran, rice bran, pomegranate, lemon, banana and orange, maximum pectinase activity of 589.0 ± 0.36 were observed in wheat bran by *Aspergillus fumigatus*. Bayoumi et al. [30] reported that maximum polygalacturonase productivity of 437.5 U/ml was obtained in the presence of 1.25 g/25 ml on *Solanum tuberosum* peels when compared to other agro-industrial wastes (*Solanum melanogena, Eichhornia crassipes* and citrus peel) by *Bacillus firmus* at 37°C for 92 hours. Deepak et al. [31] utilized fruit wastes of cashew, banana, pineapple and grape for pectinase production by *Aspergillus foetidus*. Amongst the waste material, pectinase activity was found to be at a maximum level of 0.35 U/ml in grape waste followed by pineapple waste (30.0 U/ml) at a temperature of 40°C. Palaniyappan et al. [32] investigated the production of pectinase by *Aspergillus Niger.* The results of the study revealed that the pectinase activity was found to be at the highest level of 5.17 U/ml in 1% wheat flour as a substrate. Suresh and Viruthagiri [33] studied the pectinase production using wheat bran and sugarcane bagasse as substrates by *Aspergillus Niger* and observed maximum pectinase activity of 164.15 U/ml in M2 medium with mixed substrates of 90% of wheat bran and 10% sugarcane bagasse at 96 hrs of incubation.

Anuradha et al. [34] reported that the pectin rich raw substrates like orange peel, Jack fruit rind, carrot peel and beet-root peel, the maximum pectinase production was found in Jack fruit rind (38 U/ml) followed by carrot peel (36 U/ml) bee-root peel (24 U/ml) and orange peel (16.8 U/ml) by *Aspergillus awamori*. Bhardwaj and Garg [35] evaluated the selection of the substrate for the process of enzyme biosynthesis based on the following factors. (1) They should be the cheapest agro-industrial waste with availability at any time of the year. (2) Their storage should represent no problem in comparison with other substrates and should resist any drastic effect due to exposure to other environmental conditions e.g., temperature, variation in the weather from season to season and from day tonight. The family of citrus fruits consists of oranges, kinnow, khatta, lime, lemon (Galgal), Malta, Mausami, sweet orange, etc. and they all are known to contain an appreciable amount of pectin. Besides these, other fruits like mango (*Mangifera indica*), avocado pear (*Persea americana*), guava (*Psidium guajava*), banana (*Musa sapientum*), papaya (*Carica papaya*), cashew apple (*Anacardium occidentale*), garden-egg (*Solanum nigrum* Linn.), star apple (*Crysophylum albidium*), and tomato (*Lycopersicum esculentum*) in Table 3 also contain substantial amounts of pectin having a high gelling grade.

### 2.3. Microorganisms for Pectinase Production

Initially, microorganisms such as bacteria, fungi and yeast as well as actinomycetes were extracted from the stomach of calves and baby goats. Enzymes are now produced. Micro-organism enzymes are better than animal or plant enzymes [48]. Different microorganisms are involved in the production of pectinase by using pectin as a carbon source. Pectins are degraded by several microorganisms that produce a variety of compounds and enzymes which are involved in several industrial applications. Many important bacteria, fungi and yeasts are skillful at degrading pectins substances to produce pectinases [41].

#### 2.3.1. Pectinolytic Fungi

Several *fungal* species can degrade pectic substances by producing pectinolytic enzymes. The most popular and more efficient fungi in the pectinase production are *Aspergillus Niger, Aspergillus awamori*, *Penicillium restrictum, Trichoderma viride*, *Mucor piriformis* and *Yarrowia lipolytica* have a great role in both submerged as well as solid-state fermentation for the production of various industrially important products. *Aspergillus Niger*, *Aspergillus oryzae*, and *Penicillium expansum* are the types of fungi that are generally considered safe by the United States Food and Drugs Administration are put to use in the food industry [16]. Kumari et al. [49] isolate pectinase-producing strain *Penicillium janthinellum* from the soil and has been found to produce significant amounts of an extracellular pectinase subsequently characterized as exo-polygalacturonase. The different fungal strains from vegetable wastes and screened them for their pectinolytic activity. Among them, *Tetracoccosporium* species was found to be good producers of pectinase and it showed a clearance zone of 20 mm pectinolytic activity around the colonies [50]. Khairnar et al. [51] studied the pectinase production of different strains of *Aspergillus Niger*. They observed the highest zone of clearance of pectin hydrolysis in *Aspergillus Niger* is 4.5 mm. Ten fungal isolates were isolated from municipal solid waste. Among them, a maximum zone of clearance of above 3.0 mm for pectinolytic activity was exhibited by *Penicillium chrysogenum* and *Aspergillus Niger* [52]. Different researchers have been identified very important fungi to the production of pectinase in various temperatures as well as the duration of incubation time as shown below (Table 4).

#### 2.3.2. Pectinolytic Yeasts

Kavuthodi and Sebastian [78] reported that *Saccharomyces fragilis*, *Saccharomyces thermantitonum, Torulopsis kefyr, Candida pseudotropicalis* var*, lactosa, and Candida pseudotropicalis* are types of yeast that can degrade pectin substances in the pectinase production processes. The other report also indicates additional yeast species for pectinase production, these species include *Saccharomyces* species*, Cryptococcus* species*, Aureobasidium pullulans, Rhodotorula dairenensis, Kluyveromyces marxianus, Geotrichum klebahnii*, and *Wickerhanomyces anomalus*, [13, 79].

*Wickerhamomyces anomalous* one of the classification of species *Pichia anomala* produced pectinolytic enzymes in liquid medium containing glucose and citrus pectin as carbon and energy sources. In the current studies, enzymes made by this wild yeast strain were characterized, and physicochemical properties of polygalacturonase were determined by the study of the influence of temperature and pH on its activity and stability to evaluate the application of the supernatant in the maceration of potato tissues [80].

The different investigations identified different yeast species and characterized in molecular method to the production of pectinolytic enzymes from grapes peel. Based on that identification, several species have a good potential to degrade pectin substance these species include: *Hanseniaspora* species, *Saccharomyces cerevisiae, Rhodotorula dairenensis, Candida zemplinina, Metschnikowia species, Aureobasidium pullulans,* and *Cryptococcus saitoi* [81].

#### 2.3.3. Pectinolytic Bacteria

*Erwinia* species, *Pseudomonas fluorescens, Bacillus*, *Pseudomonas*, *and Micrococcus* have a good potential to degrade pectin in the production of pectinase [20, 56, 78]. Other such as S*treptomyces* bacteria also has pectinolytic properties as reported by Ramirez-Tapias et al. [82]. *Bacillus licheniformis* has been reported as pectinolytic bacteria that were isolated from the rotten vegetable. The efficiency of *Bacillus licheniformis* to pectinase production was determined by the primary and secondary screening methods. The primary screening was carried out by the potassium–iodide flooding method and the secondary screening was carried out by fermentation. The efficiency of *Bacillus licheniformis* on the pectinase activity was recorded as 341 U/ml [83]. A newly isolated *Brevibacillus borstelensis* reported good pectinase (pectin lyase) production and characterization. The enzyme activities of *Brevibacillus borstelensis* were reported as 5.25 U/ml [84]. Soil is collected from different villages of Guntur District (Duggirala and Burripalem) from a depth of 1–15 inches to isolate desired pectinase-producing bacteria. This was used as the inoculum for the isolation of the organisms from the peel. *Bacillus pumilus* a potential pectinase-producing strain was isolated from this soil [85]. The pectinase-producing bacteria were investigated from orchard soil, at different locations of Kurukshetra, Haryana, India, using pectin agar medium at pH 7.2. Out of 109 bacterial pectinolytic isolates, isolate NV53 identified as *Bacillus* species MBRL576 produced a maximum zone of clearance after the addition of 1% cetyl trimethyl ammonium bromide and also exhibited the highest pectinase production [35]. Nine bacterial strains isolated from fruit and vegetable waste dump soil of two market areas, decayed banana, tomato and garden soil and reported that the maximum zone of clearance was observed in the bacterial isolate *Streptococcus* species 5 cm followed using *Staphylococcus aureus anaerobius* 5 cm [86]. Two bacterial strains isolated from rotten oranges and reported that the largest pectinolytic zone of 25 mm was observed by two bacterial strains *Staphylococcus aureus* and *Bacillus cereus* [87]. Different research works show that, among different bacterial isolates screened for pectinolytic properties *Bacillus* strains were selected as the most potent enzyme producers [56, 88].

### 2.4. Factor Affecting Pectinase Production

Various Factors affect microbial pectinase production.

#### 2.4.1. Effect of pH on Pectinase Production

The effects of pH on pectinase production have been reported by various researchers. Torimiro and Okonji [54] investigated the pectinase production by *Bacillus* species. Their report tried to the optimized effect of pH on the production of pectinase, the range of optimized pH was 4–10. But the maximum amount of pectinase was recorded at pH 7. The production and optimization of pectinase were carried out by *Bacillus* species MFW7 using Cassava as substrate, the optimization of this pectinase production was included various ranges of pH 3.5, 4.5, 5.5, 6.5, 7.5, 8.5 and 9.5, among that wide range of pH, the maximum pectinase activities were observed at pH of 6.5 as reported by Kumar et al. [89]. Another investigation showed pectinase produced from *Chryseobacterium indologenes* strain SD. This production was optimized with different ranges of pH 5–9 in 0.5 intervals, but the highest production of pectinase was obtained at pH 7.5 as investigated by Roy et al. [90]. The production of polygalacturonase was optimized by different ranges of pH by using *Bacillus sphaericus.* The ranges of pH were 4.4, 5, 5.6, 6.2, 6.8 and 7.4 in 0.6 intervals. From that pH variation, the maximum activities of polygalacturonase were obtained at 6.8 as reported by Jayani et al. [91]. The effect of pH on pectinase production was also optimized by using *Bacillus* species FW5 and *Erwinia* species FW2. The ranges of pH were 5–9. Among those various ranges of pH, the maximum production of pectinase was obtained at 7 by both *Bacillus* species FW5 and *Erwinia* species FW2 as reported by Mehta et al. [61].

#### 2.4.2. The Effect of Temperature

Different researchers obtained maximum pectinase production by various bacterial species in different temperature ranges. The polygalacturonase was produced at different temperatures starting from 25–50°C in five-degree Celsius intervals. From this temperature, the maximum polygalacturonase was produced at 30°C by *Bacillus sphaericus* [91]. The maximum amount of production of polygalacturonase was produced by *Enterobacter tabaci* NR1466677. This study was carried out starting from 20–45°C. The optimum temperature of this enzyme production was observed at 35°C [92]. The maximum pectinase produced by *Erwinia* species FW2 within various temperature ranges. This temperature range was 20–65°C. Among those temperatures, the maximum pectinase production was observed at 37°C as reported by Mehta et al. [61]. The alkaline pectin lyase is produced by a newly isolated *Brevibacillus borstelensis* (P35). This alkaline pectinase was produced in the temperature range of 20–100°C. The maximum amount of this pectin lyase was observed at 60°C as reported by Demir et al. [84]. An extracellular Pectinase was also produced by newly isolated *Bacillus subtilis* strain and the maximum total activity of pectinase from *Bacillus subtilis* grown in medium including pectin as a carbon source at 37°C as reported by Mercimek takci and Turkmen [93]. Polygalacturonase is one part of pectinase produced by pectinolytic bacteria *Bacillus licheniformis* strain GD2. The production of polygalacturonase was produced in the three temperature ranges of 45–65°C and among these temperatures, the maximum amount of polygalacturonase activities appeared at 45°C [94]. The various *Bacillus* species have been reported as they can produce pectinase within various temperature ranges. The maximum activities of pectinase were recorded by *Bacillus firmus and Bacillus endophyticus* at the temperature of 37°C. The *Bacillus coagulant* and *Bacillus vietnamisis* were produced the maximum amount of pectinase activities at the temperature of 30°C as reported by Khan and Barate [95]. A different study shows that *Erwinia* species can produce polygalacturonase. The highest amount of polygalacturonase activities has been reported that were investigated in different temperature ranges starting from 20–45°C. But the maximum polygalacturonase activities were recorded at 35°C by *Erwinia carotovora* MTCC1428 as reported by Kothari and Baig, [58].

#### 2.4.3. The Effect of Fermentation Times

The maximum production of pectinase from different microorganisms varies from time to time. The *Bacillus* species MFW7 produced a significant amount of pectinase after 96 hours of incubation in fermentation medium reported by Kalaichelva [89] and *Erwinia carotovora* MTCC1428 produced the maximum amount of polygalacturonase activity at the end of 72 hours fermentation time in liquid state fermentation condition [58]. The highest activities of polygalacturonase were observed after 120 hours of fermentation time by using *Bacillus* species [96]. The other bacterial isolate K6 was identified as *Chryseobacterium indologenes* strain. This isolate produces maximum extracellular pectinase at the end of 72 hours of incubation time [90]. The alkaline pectinase is also produced by the *Cocci* species. This species produced the maximum amount of pectinase after 72 hours fermentation times [97]. The maximum pectinase was produced using *Erwinia* species FW2 and *Bacillus* species FW5. Those two different species produced the maximum amount of pectinase after the end of 96 hours of fermentation time as reported by Mehta et al. [61].

#### 2.4.4. The Effect of Substrate Concentration

As the concentration of pectin varies, it affected the production of pectinase. The highest amount of pectinase activities were observed at 0.8% of pectin concentration. This maximum activity of pectinase was carried out by taking various concentration ranges (0.1–1%) in 0.1% intervals of pectin concentration as investigated by Khan and Barate, [95]. Other studies indicated that as the concentration of pectin increased, the activity of pectinase increased up to optimum concentration and after the optimum concentration, the activity of pectinase was decreased. Among the various pectin concentrations ranges 0.1%, 0.2%, 0.5%, 1%, and 1.5%, the maximum amount of pectinase activities were recorded at 0.5% of pectin concentration and decreased after this concentration as reported by Mehta et al. [61]. Polygalacturonase is one part of pectinase that shows a good activity by different citrus pectin concentration ranges 0.25%, 0.5%, 0.75%, 1%, 1.25%, and 1.5%. The highest amount of polygalacturonase activities was recorded at 1.25% of citrus pectin concentration and the activities of polygalacturonase were decreased after that optimum pectin concentration as reported by Jayani et al. [91]. Polygalacturonase can produce from glucose, sucrose, galactose and soluble starch as a carbon source by *Enterobacter aerogenes* NBO2. This enzyme has been produced with different concentration of carbon sources (0.5%, 1%, 1.5%, 2%, 2.5% and 3% w/v). From those various concentrations of carbon source, the maximum amount of polygalacturonase was recorded at 1% of each carbon source as investigated by Darah et al. [98]. The highest Exo and endo pectinase activity of 0.79 U/ml and 0.01 U/ml was reported in sugar beet as substrate using *Aspergillus Niger* [99]. Okafor et al. [100] during their investigation on two pectinase-producing fungal isolates, *Aspergillus Niger* and *Penicillium chrysogenum* using the different agro-wastes, including pineapple peel, orange peels, sawdust, sugarcane pulps and wheat bran, as the sole carbon source reported the highest pectinase activity of 350.28 and 478.25 Uml-1 protein using *Aspergillus Niger* and *Penicillium chrysogenum,* respectively, in wheat bran as sole carbon source. From various agricultural waste and agro-industrial byproducts (banana peel, wheat bran, sugar cane bagasse, and orange bagasse), the best substrate for PG production by *Penicillium* species was found to be in orange bagasse with enhanced enzyme production of 64.5 U/mg followed by wheat bran 53.6 U/mg [101].

## 3. Industrial Applications of Microbial Pectinases

**Figure 4:**
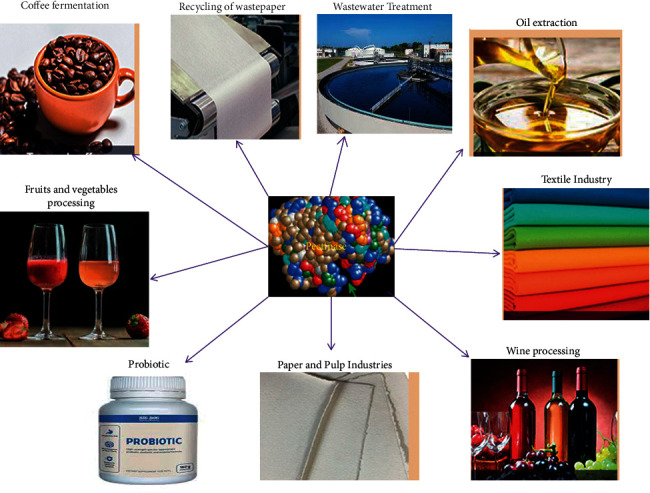
Various applications of pectinases [21, 26, 102].

Applications studies with pectinases are ongoing in global research fields to obtain maximum fastened activity with enzymes. The wide application of pectinase is attributed to its increasing global demand. The application of pectinolytic enzyme is varied according to the availability of physical conditions. Pectinases have been used in several conventional industrial processes, such as textile, plant fiber processing, tea, coffee, and oil extraction and treatment of industrial wastewater, containing pectinaceous material in Figure 4.

### 3.1. Textile Processing

Pectinase, in combination with other enzymes such as amylase, lipase, cellulase, and hemicellulase, has been used in the textile industry to remove sizing agents from cotton, substituting the usage of harsh chemicals [103]. Different combinations of enzymes, such as cellulose with pectinase and cellulose with pectinase and protease, have been utilized for the bioscouring of cotton to achieve effective whiteness and absorbency of the textile fabric [104]. The use of enzymes such as pectinases in conjunction with amylases, lipases, cellulases and other hemicellulolytic enzymes to remove sizing agents has decreased the use of harsh chemicals in the textile industry, resulting in a lower discharge of waste chemicals to the environment, improving both the safety of working conditions for textile workers and the quality of the fabric [105].

### 3.2. Fruits and Vegetable Processing

Pulp treatment, fruit juice extraction, and clarity are all factors in the use of microbial pectinases in the fruit and vegetable industry. Pectinases contribute to the reduction of viscosity, the clarity of juice, and the maceration of vegetables, as well as the reduction of fermentation time [104, 106, 107]. In the fruit and vegetable juice industry, pectinase is widely used. These industries commercially produce a variety of juices, including sparkling clear juices, cloudy juices, and unicellular products, to selectively hydrolyze middle lamella polysaccharides to safeguard plant cell integrity [103]. Since the pectinolytic enzyme is one of the upcoming enzymes, most fruit industries are used in fruit juice processing. Due to the presence of pectin polysaccharide in fruit juice, the fruit juice is naturally cloud [108]. By nature; any fruits have a high concentration of pectin. This high concentration of pectin leads to the colloid formation in the juice, which leads to creating problems in the processing of clear fruit juices. The appearances of cloudiness in fruit juices also lead to a problem in the market. The traditional processes of extracting fruit juice are also not attractive and consume huge energy. Due to this reason, pectinase has a great role in the production, extraction and extraction of fruit juices [90]. The use of the enzymatic solution for the treatment of fruits and vegetable mash afforded a high juice extraction and a pulp with good pressing characteristics [96]. Pectinase is used in the fruit juice industry before clarifying to avoid pectin-protein flocculation and reduce viscosity. To improve permeation flux in microfiltration, ultrafiltration, and reverse osmosis, the early treatments of fruit juice with the addition of pectinase have been researched in large quantities [55]. According to the report of Ajayi et al. [109]; pectinase enzymes that obtained from deteriorated fruits as substrate was used in the clarification of apple juice from various apple fruits with different volume of pectinase to compare with the corresponding volume of water and commercial pectinase where applied in the juice clarification process. Pectinase lowers the viscosity of fruit juice during the clarifying process by degrading the pectin material in the juice and improving the pressing ability of the pulp, while simultaneously breaking down the jelly structure and increasing the yields of fruit juice. The refinement of vegetable fibers during the starch manufacturing process, such as the curing of coffee, cocoa, and tobacco, canning of orange segments, and extracting sugar from date fruits, is another important feature of pectinase enzymes in industrial processes [48]. Pectinases improve fruit juice production by reducing blurred vision and breaking pectic structures [110]. The use of pectinases in the preparation of fruit juice encourages the liberation of phenolic compounds from the fruit skin [111].

### 3.3. Wine Processing

Pectinolytic enzymes' primary roles in the winemaking process are to aid in extraction, maximize juice yield, facilitate filtration, and intensify flavor and color [106]. The use of pectinases in winemaking accelerates maceration, enhances juice extraction yield, speeds up filtration, and improves flavor and color. Before inoculating the alcoholic fermentation, the fruits were macerated with pectinases. This technique improves the wine's quality [111]. The addition of pectic enzymes to the crushing of fruits during the winemaking process enhances the volume of free-flow juice and reduces pressing time. It also aids in the filtration and clarity of juice as well as also improves the chromaticity and stability of red wines [112].

### 3.4. Coffee and Tea Fermentation

**Figure 5:**
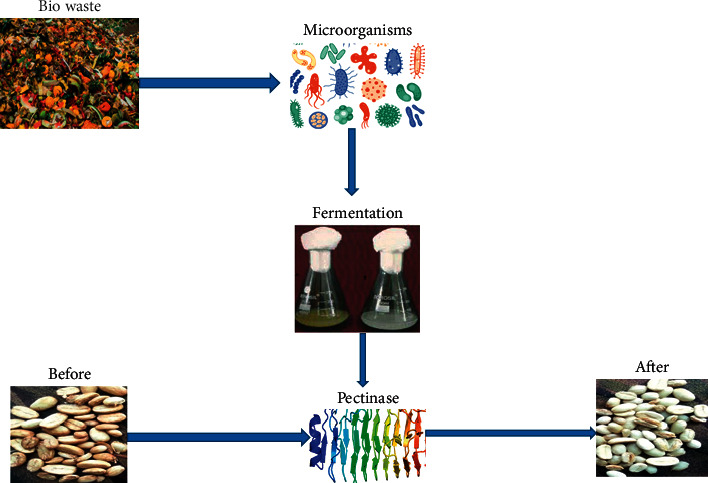
Pectinase for mucilage removal of coffee beans [17].

In the process of tea fermentation, instant tea powder have a great role to make drinking tea. This instant tea powder has a huge concentration of pectin because it is made from leaves. The preparation of tea by using this powder leads appearance of foam formation on tea due to the high concentration of pectin. Pectinase such as Polygalacturonase is used in the tea process to destroy the foam-forming property of instant tea powders by destroying pectins, increasing the quality of tea, color changes and highly valuable in the market [13, 113]. Pectinase is also used in the coffee fermentation process. The coffee bean has had covers that surrounded its internal structures these hardcovers of coffee beans are called mucilage. The mucilage also has viscous and gelatinous properties that are not comfortable to make drinkable coffee. During the process of the alkaline pectinase is used to remove mucilage coat from the coffee bean before using the coffee bean [113]. Coffee is fermented with pectinolytic microbes to remove the mucilage coat from the beans and to improve tea fermentation and froth-producing properties [21]. Alkaline pectinases have been employed in tea fermentation to degrade pectins and remove the mucilaginous layer from coffee beans [17] in Figure 5, preventing the foaming of instant tea granules [114].

### 3.5. Oil Extraction

Pectinase and other cell wall degrading enzymes (CWDE) have been widely explored for oil extraction from various sources such as flaxseed, olives, dates, and so on [104]. Citrus oils, such as lemon oil, can be extracted with pectinases because these enzymes disrupt pectin's emulsifying properties, which prevent oils from being removed from citrus peel extracts [14, 105].

### 3.6. Paper and Pulp Industries

The use of chlorine-containing bleaching compounds in the paper and pulp industry produces toxic, mutagenic, and bioaccumulating organochlorine byproducts. These are the source of significant disruption in the ecosystem. The use of pectinase in this case is to avoid the toxicity of chlorinated compounds in the ecosystem [107, 111]. Pectinases are used in the papermaking process to depolymerize galacturonic acid polymers, reducing the cationic requirement of pectin solutions [14, 114]. In the paper and pulp industry, sheet formation is a critical process that is affected by the presence of pectins in the pulp, which causes yellowing of the paper [111, 112].

### 3.7. Recycling of Waste Paper

Environmental risks are created by chemical deinking; however, enzymatic deinking minimizes pollution risks, energy consumption, disposal problems, and enhances performance. During the deinking process, a group of enzymes (pectinases, hemicellulases, cellulases, and ligninolytic enzymes) is used. Enzymes alter the bonding properties of ink and fiber, resulting in the removal of ink from the surface of the fibers during washing [111].

### 3.8. Wastewater Treatment

For treatment of wastewater from citrus processing industries, various processes have been investigated, which include: physical dewatering, spray irrigation, chemical coagulation, direct activated sludge treatment and chemical hydrolysis followed by methane fermentation. These processes have low efficiency due to chemical resistance of the pectic substances, high treatment cost, long treatment periods and complexity of the process farces [12]. Vegetable food processing industries release wastewater as by-products. These wastes have pectic substances because naturally, those vegetables are rich in pectic substances. Pretreatment of these wastewaters with pectinolytic enzymes facilitates removal of pectinaceous material and renders it suitable for decomposition by activated sludge treatment, [113]. Pectin is released by the vegetable food processing industry as a by-product, which contains wastewaters. The addition of pectinolytic enzymes to these wastewaters enhances the removal of pectinaceous material and makes them amenable for decomposition by activated sludge treatment [21]. Pectinases are enzymes that are used to remove pectin from wastewaters before they are treated. The use of pectinolytic organisms during the treatment of activated sludge is an environmentally beneficial, cost-effective, and time-saving process [111].

### 3.9. Prebiotics/Functional Foods

A prebiotic is a fermented food that allows for specific changes in the gut microbiome's makeup and/or activity to enhance the host immune system [107, 115]. Pectin and pectin-derived oligosaccharides (PDO) are emerging as excellent candidates in new generation prebiotics. It has been observed that intestinal bacteria ferment methylated pectin to form short-chain fatty acids (SCFA) such as acetate, propionate, and butyrate, which are beneficial to health [107]. Pectinase is used to make functional food components and nutraceuticals, as well as to boost food's antioxidant potential [116].

## 4. Challenges and Future Opportunities of Applying Microbial Pectinase in Industry

Enzymes have a major impact on practically every industrial area (for example, food, feed, pharmaceuticals), and as a result, the market for industrial enzymes is rapidly expanding to meet the ever-increasing demand of consumers [117]. However, the stability of enzymes and the cost of enzymes lead to a delay in their advancement in industrial sectors. For commercialization, the enzyme's stability against severe temperatures, adverse pH environments, and organic solvents is critical. Enzymes' limited resistance to intense industrial conditions limits their application in commercial processes [103]. Several approaches can be used to optimize pectinase production; however, because enzymes are unstable, the cost of broad applicability is higher [104, 117]. Thermophilic enzymes are gaining attention in research because temperature control during large-scale fermentation processes is challenging and expensive [107]. The cost viability of producing pectinase from selective microorganisms and implementing environmental conditions is one of the most important factors [118]. Pectinases can be used in a variety of industrial processes to improve the quality and quantity of final products. In this approach, it's critical to look into the production process and physicochemical properties of novel enzymes [119]. To further reduce the overall cost, research should be conducted in the area of immobilization of pectinase enzyme for reusing purposes [120]. Genetic engineering is a far more efficient option because the changes are completely controlled. This process involves taking the relevant gene from the microorganism that naturally produces a particular enzyme (donor) and inserting it into another microorganism that will produce the enzyme more efficiently (host) [22]. More study is needed to find strains that produce pectinase in combination with other enzymes, and the exact mix is needed for each application. This will reduce the cost of production for a specific application significantly. The emphasis of future pectinolytic research should be on elucidating the molecular mechanisms that regulate enzyme secretion as well as the mechanisms of action of distinct pectinolytic against various agro-industrial pectic substrates. In this way, well-designed studies can provide important tools for manipulating microbes to produce high quantities of efficient and cost-effective enzymes. Enzymology, molecular biology, and screening techniques have advanced, allowing the textile industry to develop new enzyme-based technologies that are more environmentally friendly. Pectinases appear to be able to perform all processes in the future. Pectinases have great attention in industrial applications such as the textile industry and fruit processing industries, oil extraction, coffee and tea fermentation. Finally, it is concluded that the role of pectinases in various industrial processes has been discovered to be curiously recognizable, with promising outcomes. From the extensive research, it is clear that pectinolytic enzymes have been considered as an imperative for the significant development or improvement of enzymes to industrial applications. Thus, the successful completion of this approach for the use of microbial pectinase, research should focus on protein engineering to obtain more robust and versatile pectic enzymes, as well as the optimization of production processes using new strains.

## Figures and Tables

**Table 1:** Type of pectic substances and its description.

S/No	Type of pectin	Description	Source
1	Pectinic acid	Having the various amounts of methoxyl and under suitable conditions can form a gel with sugar	Nawaz et al., [16]
2	Pectic acid	Galacturonans have no methoxyl group, have the property of solubility and its normal or acid salt is called pectate	Oumer [14]
3	Protopectin	Water-insoluble parts of pectic substances, mostly present in unripe fruit and it degrading by protopectinase	Nawaz et al. [16]
4	Pectin	75% of the carboxyl groups of the galacturonate units are esterified with methanol	Oumer [14]

**Table 2:** Percentage of pectin in some vegetables and fruit [19].

S/No	Fruit/vegetable	Tissue	Pectin substance (%)
1	Banana	Fresh	0.7–1.2
2	Peaches	Fresh	0.1–0.9
3	Strawberries	Fresh	0.6–0.7
4	Lemon	Fresh	0.63
5	Peas	Fresh	0.9–1.4
6	Carrots	Dry matter	6.9–18.6
7	Orange pulp	Dry matter	1.4–2.8
8	Avocado peel	Dry matter	3.4–5.2
9	Potatoes	Dry matter	1.8–3.3
10	Tomatoes	Dry matter	2.4–4.6
11	Sugar beet pulp	Dry matter	10–30
12	Apple	Fresh	0.6–1.6

**Table 3:** Agro-industrial waste as substrates for pectinase production by using microorganisms.

S/No	Agricultural residues	Microorganisms	Types of enzymes	Fermentation states	Sources
1	Wheat bran	*Aspergillus giganteus, Aspergillus sojae.*	Polygalacturonase	SSF	Demir and Tari [36], Heerd et al., [37], Anand et al. [38], Ortiz et al., [39]
2	Rice husk and rice bran	*Aspergillus fumigatus*	Polygalacturonase	SSF	Wong et al., [40], Tai et al., [41]
3	Papaya peel	*Aspergillus tubingensis*	Pectin methylesterase and polygalacturonase	SSF	Maran and Prakash [42], Patidar et al. [28]
4	Mango peel	*Aspergillus foetidus, Enterobacter* spp.	Pectin methylesterase	SSF and SmF	Cheok et al. [43], Chandra et al. [44]
5	Sugarcane bagasse	*Aspergillus Niger*	Pectinase	SSF	Patidar et al. [25]
6	Sunflower head	*Aspergillus Niger*	Pectinase	SSF and SmF	Patidar et al. [25]
7	Banana peel	*Aspergillus terreus, Aspergillus Niger*	Pectinase	SSF	Sethi et al. [45], Barman et al. [46]
8	Algal biomass	*Bacillus licheniformis*	Pectinase	SmF	Pervez et al. [47]
9	Grape pomace	*Aspergillus awamor*	Polygalacturonase	SS	Patidar et al. [25]
10	Strawberry pomace	*Lentinus edodes*	Polygalacturonase	SSF	Patidar et al. [25]

**Table 4:** Optimization of important microorganisms in pectinase production.

Bacteria								
S/No		Microbial specie*s*	Type of pectinase	pH	Temperature oC		Incubation time	Sources
1		*Bacillus* sp.	PME	6	60		—	Karbalaei and Rastegari [53]
2		*Bacillus stearothermophilus*	Pectinase	7.5	60		36 h	Torimiro and Okonji [54]
3		*Bacillus cereus*	Pectinase	8	50		36 h	Torimiro and Okonji [54]
4		*Bacillus firmus*	PG	7	50		30 min	Roosdiana et al. [55]
5		*Bacillus. Mojavensis*	Pectinase	8	60		24 h	Sohail and Latif [56]
6		*Bacillus pumilus*	Exo pectinase	8	30		6 h	Tepe and Dursun [57]
7		*Erwinia carotovora*	PG	5.2	35		72 h	Kothari and Baig [58]
8		*Erwinia carotovora*	PL	7	35		—	Zucker and Hanki [59]
9		*Pectobacterium carotovora*	PL	8.5	50		—	Masuria and Nerurkar [60]
10		*Erwinia* spp.	Pectinase	7	37		96 h	Mehta et al. [61]
11		*Erwinia carotovora*	PG and PL	10	35		—	Sittidilokratna et al. [62]
12		*Erwinia chrysanthemi*	PG	10	37		—	Sittidilokratna et al. [62]
13		*Erwinia carotovora*	*PG*	10	50		—	Jayani et al. [11]
14		*Erwinia chrysanthemi*	Pectinase	5–9	50		—	Jayani et al. [11]
15		*Bacillus subtilis*	—	5	50		—	Prajapati et al. [63]
16		*Bacillus tropicus*	—	9	37		72 h	Thakur et al. [64]
**Fungi**								
17		*Penicillium chrysogenum*	PG	6.5	50	5 days		Banu et al. [52]
18		*Aspergillus oryzae*	Pectinase	—	30	2 days		Thangaratham and Manimegalai [65]
19		*Aspergillus flavus*	Pectinase	5.5	35	7 days		Thangaratham and Manimegalai [65]
20		*Moniliella* sp	PG	4.5	55	15 days		Martin et al. [66]
21		*Trichoderma harzianum*	Pectin lyase	7	40	3 days		Nabi et al. [67]
22		*Aspergillus sojae*	PG	—	30	8 days		Heerd et al. [38]
23		Aspergillus Niger ABT-5	Pectinase	6	30	3 days		Abdullah et al. [68]
24		*Aspergillus sojae*	PG	6	37	4 days		Demir and Tari [37]
25		*Schizophyllum commune*	Pectin lyase	6	35	1 day		Mehmood et al. [69]
			Methylesterase	6	35	3 days		Mehmood et al. [70]
			Polygalacturonase	4	45	5 days		
26		*Fusarium proliferatum*	Polygalacturonase	3.6	43.4	—		Junior et al. [71]
27		*Aspergillus Niger*	Polygalacturonase	7.5	40	3 days		Adedayo et al. [72]
28		*Aspergillus flavus*	Polygalacturonase	7.7	40	3 days		
**Yeast species**								
29	*Wickerhanomyces anomalus*		Polygalacturonase	4.5	40–50	—		Martos et al. [73]
30	*Wickerhamomyces anomalus*		Polygalacturonase	—	—	8 hr		Martos et al. [73]
31	*Saccharomyces cerevisiae (*strain KNU18Y12 and KNU18Y13)		Pectin methylesterase (PME)	—	28	48 hr		Haile and Kang [74]
32	*Kluyveromyces marxianus* CCT 3172 and *P. anomala* S16		Polygalacturonase	5.5	40	—		Masoud and Jespersen [75]
33	*P. kluyveri* S13Y4		Polygalacturonase	5	50	—		
34	*Kluyveromyces marxianus* CCT 3172*, P. anomala* S16 and *P. kluyveri* S13Y4		Polygalacturonase	6	30	—		
35	*Filobasidium capsuligenum*		Pectinase	4.5	40	2 hr		Merín et al. [76]
36	*Kluyveromyces marxianus* NRRL-Y-1109		Pectinase	6	30	48 hr		Oskay & Yalcin [77]

## Data Availability

No data were used to support this study.
